# Deciphering Platelet Kinetics in Diagnostic and Prognostic Evaluation of Hepatocellular Carcinoma

**DOI:** 10.1155/2018/9142672

**Published:** 2018-06-27

**Authors:** Bibek Aryal, Munekazu Yamakuchi, Toshiaki Shimizu, Jun Kadono, Akira Furoi, Kentaro Gejima, Teruo Komokata, Teruto Hashiguchi, Yutaka Imoto

**Affiliations:** ^1^Cardiovascular and Gastroenterological Surgery, Graduate School of Medical and Dental Sciences, Kagoshima University, Kagoshima 890-8520, Japan; ^2^Department of Laboratory and Vascular Medicine, Graduate School of Medical and Dental Sciences, Kagoshima University, Kagoshima 890-8520, Japan; ^3^Department of Surgery, Kirishima Medical Center, Kirishima 899-5112, Japan; ^4^Department of Surgery, Kagoshima Medical Center, National Hospital Organization, Kagoshima 892-0853, Japan

## Abstract

Liver pathophysiology can, directly and indirectly, impose morphological or biochemical abnormalities of the platelets. Conversely, platelets are also able to regulate the promitogenic and profibrotic signals on liver pathobiology. Platelet contribution to the liver pathophysiology is typically facilitated by the platelet-derived growth factors that are sequestered in different subsets of alpha and dense granules, and the release of these growth factors is synchronized according to the stage and type of liver disease or injury. Thus, platelets harbor clinically relevant information with potential diagnostic and prognostic implications in liver diseases. Hepatocellular carcinoma (HCC) largely influences the platelet kinetics, and a growing body of evidence has recognized its association with HCC occurrence or prognosis. This narrative review summarizes the progress made on implicating platelet as a diagnostic and prognostic tool for HCC; the review also dissects the contradictory results from earlier studies and reflects how combining platelet-based information may enable more reliable test for diagnostic and prognostic evaluation of HCC.

## 1. Introduction

Hepatocellular carcinoma (HCC) is the leading cause of cancer deaths worldwide with the highest incidence in Asia and Africa, where the endemic prevalence of hepatitis B (HEP-B) and hepatitis C (HEP-C) strongly predisposes to the development of chronic liver disease (CLD) and consequent occurrence of HCC. In 2015 alone, HCC accounted for estimated 470,000 deaths globally [1]. The incidence rate and liver cancer-related death are expanding to other parts of the world, including North America, Latin America, and central Europe [2]. Apart from HEP-B and HEP-C, a range of risk factors for the development of HCC has been identified, and a majority of the patients develop fibrosis or cirrhosis before cancer progression [3]. The life expectancy or the overall survival (OS) in patients is still discouragingly poor with a mean survival of 6–20 months [4]. Despite tremendous progress in the diagnostic and therapeutic approach, the OS has not been impressively prolonged yet.

Transplant or curative resection of HCC offers the best outcome in a selected group of patients; however, the long-term recurrence-free survival (RFS) rates are limited to 40 percent or less [4–6]. Although several systems have been proposed to predict the prognosis, there is still no single system that holds the best predictive solution in HCC patients [7, 8]. Thus, the close surveillance is not only crucial in patients with underlying cirrhosis but also equally important to predict or identify HCC recurrence after the curative treatment.

There is already a pile of evidence that highlights the role of platelets in liver homeostasis and inflammation [9, 10]. Platelet kinetics appears to be affected by pathological conditions of the liver providing opportunities to use platelet as diagnostic, prognostic, or predictive tools in HCC patients. Besides their traditionally recognized role in hemostasis and coagulation, blood platelets, the circulating anucleated cells, have emerged as key players in systematic and local response to tumor progression [7, 8, 11, 12]. This narrative review summarizes the recent advances in platelets dynamics and its potential diagnostic and prognostic implications in patients with HCC.

## 2. Literature Search

A literature search was performed for the current study on the benchmark of searches for a narrative review article. The electronic search comprised mainly two databases, PubMed and Google Scholar, and used two search terms: “platelet and hepatocellular carcinoma”. Besides, additional references were obtained by manual search. The inclusion criteria were all related articles displayed in PubMed and Google Scholar. Articles without full text and not in English were excluded.

## 3. The Orbit of Hematology (Platelets) and Hepatology

Platelets, nonnucleated cell elements, born out of the cytoplasmic fragment of bone marrow megakaryocytes, inherit some very important properties of typical cells. These small fragments, sometimes called pollutants or even cell debris, are equipped with the sense to discriminate several types of danger signals and adjust their response [13]. This response is, in general, physiological, a platelet's routine duty of patrolling the cracks and erosions in the vascular endothelium, and fixing the breaches or the leakage. After being known for the role in hemostasis and thrombosis, they were recognized to interact with cells other than the vascular endothelial cells; platelets were then pulled to the arena of inflammation, immunity, and carcinogenesis [11, 12, 14, 15].

A closely interlaced relationship between hemostasis and hepatology brings platelets always inside the ring during liver injury or inflammation. Platelets accumulate in the injured liver and interact with liver cells in a disease-specific and stage-specific manner [9]. Platelets are able to enter hepatic sinusoidal circulation, influencing effector cell recruitment and activation. Platelets are known to interact with different hepatic cells, and these cellular interactions can result in the release of a range of bioactive proteins including cytokines, chemokines, and growth factors from platelet granules. By releasing these bioactive growth factors or cytokines, platelets participate in hepatic pathological or physiological events, from liver repair, regeneration, and fibrosis to carcinogenesis [9, 10, 16].

## 4. Influence of Platelet Count

A majority of HCC is diagnosed in patients with underlying cirrhosis or CLD. Distortion of the normal liver architecture in patients with cirrhosis increases resistance to blood flow, resulting in portal hypertension, splenomegaly, and resultant thrombocytopenia. Thus, the platelet count has its own classical merits in predicting cirrhosis [17, 18] and its outcome or in assessing the severity of fibrosis [19, 20]. Studies have now elucidated various pathways on the platelet kinetics and its consumption in liver diseases and have broadened the understanding of thrombocytopenia in liver disease [21].

Association between platelet count and prognosis is established in different solid tumors. A reduced risk for cancer metastasis with aspirin administration at an antiplatelet dose reported recently corroborates the long-established relationship between thrombocytosis and cancer progression [22, 23]. Moreover, the paraneoplastic thrombocytosis is mediated closely by hepatic thrombopoietin synthesis that is augmented in response to excessive tumor-derived interleukin-6 [22].

Various studies have explored the link between platelet count and HCC; in a recent meta-analysis, Pang et al. clinched a conclusion suggesting an overall lower pretreatment platelet count as a risk factor for the overall and tumor-free risk of death [24]. Yılmaz et al. suggest platelet count as an indicator of risk for HCC development in patients with cirrhosis rather than a tumor marker itself [25]. In a large prospective cohort, thrombocytopenia was observed in 40.7% of patients with smaller tumors, but only in 11.3% of patients with larger tumors, and the platelet count was significantly associated with the tumor size [26].

Platelet count has also pulled some attention for its predictive relevance in HCC recurrence after curative resection. Several studies suggest thrombocytosis as a predictive factor for HCC recurrence after liver resection; however, there is also contrasting evidence that reports thrombocytopenia as a risk factor for postresection HCC recurrence. In one recent study, the postresection recurrence rate was stratified by the postresection platelet count, demonstrating an inverse relationship between the recurrence and postresection platelet concentration [27]. In patients who underwent hepatic resection, the presence of a lower level caused a 0.67-fold increase in the risk of overall mortality and a 0.44-fold increase in the risk of disease-free death in comparison with a higher level [28]. In our previous study, we also observed the postresection depletion of platelet count in patients who developed early HCC recurrence [29].

## 5. Platelet to Lymphocyte Ratio

Platelet-lymphocyte ratio (PLR), an inflammation-based score, has lately gained a substantial attention as a potential prognostic marker in different solid tumors. A wealth of studies has investigated the prognostic significance of PLR in HCC; the conclusions drawn from the studies are distinctly contradictive. A positive association was observed between high PLR and poor prognosis in patients with transarterial chemoembolization (TACE); high PLR was also able to predict OS [30, 31]. In another study conducted in patients who underwent surgical resection for HCC, PLR failed to predict both OS and RFS [32]. The results obtained from CHB-related HCC patients after TACE showed no evident relationship between the PLR and OS [33]. Although, in a recent meta-analysis by Zhao et al., high PLR distinctly predicted shorter OS in patients undergoing surgery for HCC, no obvious relationship was observed between the PLR and RFS [34].

Besides PLR, the prognostic value of platelet, neutrophil, and lymphocyte ratio (PNLR) has also been examined. In a retrospective study conducted in patients with Barcelona clinic liver cancer (BCLC) stage A HCC, increased postoperative PNLR was associated with higher incidence of postresection recurrence and poor OS [35].

## 6. Intraplatelet Serotonin: Wrapped in an Enigma?

A large portion of serotonin, secreted by enterochromaffin cells of the intestine, is taken up by platelets; thus, circulating platelets act as a warehouse for serotonin. Platelets sequester about 95% of total plasma serotonin in their dense granules [9] and release it in response to various stimuli [36]. An overwhelming body of evidence has elucidated serotonin's crucial role in regulating hepatic function and response to injury; it interacts with both hepatocytes and hepatic stellate cells and mediates vasoconstrictive action on the sinusoidal circulation [37]. Besides its promitogenic and profibrotic effect on liver, platelet serotonin has also gained an extensive attention for its regenerative property after liver injury [38, 39].

Growth promoting effect of serotonin was found in different liver cancers including HCC. Serotonin promotes survival and growth of HCC cells by activation of serotonin receptor 2B. Two serotonin receptors 1B and 2B were found overexpressed in the liver of patients with HCC [40]. Serotonin signaling plays certain roles in determining the balance between fibrogenesis and regeneration in CLDs, which may be implicated in the carcinogenesis of HCC. Platelet-hepatocyte interaction is presumed to mediate the initiation of mitogenic signaling, through the intraplatelet (IP) growth factors; thus IP serotonin is considered as one of the crucial stimulants of liver cell proliferation.

Monitoring IP serotonin served as a sensitive marker and provided relevant clinical information for diagnosis of some tumors [41]. Elshayeb et al. observed elevated serotonin concentration in patients with HCC and found an enhanced predictability of alpha-fetoprotein (AFP) when combined with serotonin suggesting serotonin as a novel marker for HCC diagnosis [42]. Another study proposes the combined use of serotonin and protein induced by vitamin K absence-II (PIVKA-II) as a potential marker to screen for HCC in cirrhotic patients with chronic hepatitis C (CHC) [43].

Padickakudy et al. have recently discovered a bivalent property of platelet-sequestered serotonin in liver regeneration and HCC recurrence; high perioperative platelet serotonin concentration was associated with an increased incidence of early tumor recurrence, and excessively low platelet serotonin levels correlated with a higher rate of morbidity in patients who underwent liver resection for liver tumors [44]. Beyond the accepted paradigm of mitogenic property of serotonin in tumor cells, some opposing evidence also argues on its antitumor property [45]. Deb et al. observed depleted IP serotonin in patients with cervical cancer and found that platelets from cancer patients released more serotonin on thrombin stimulation than the control group [46]. In a homogenous prospective cohort of patients with HCC, we found a postresection exhaustion of IP serotonin was able to predict early HCC recurrence [29]. The study conducted by Padickakudy et al., with a highly standardized preparation of platelet, and our study, however, referred to different postresection time points; the former study monitored IP serotonin during early postoperative period whereas we measured it after 4 weeks of liver resection. It has also been suggested that pleiotropic action of IP serotonin on the liver is determined by the cellular and cytokine microenvironment specific to the stage of liver injury [9]. Platelet-derived serotonin further exemplifies the complexity and diverse roles of platelets in liver pathophysiology. With this multifaceted association of IP serotonin in liver pathophysiology and cancer, a solid evidence for recommending IP serotonin in HCC diagnostic and prognostic is far from complete.

## 7. Platelet Vascular Endothelial Growth Factor

A major pool of vascular endothelial growth factor (VEGF), a key driving force for physiological and pathological angiogenesis, is largely sequestered in the blood platelets [47]; thus, platelets act as a major physiological transporter of VEGF in the circulation. Serum VEGF reflects both plasma held and IP concentrations. Since plasma contains a substantially lower concentration of VEGF, platelets are the major source of serum VEGF. Several studies have demonstrated a direct correlation between platelet count and serum VEGF [48, 49], and a significant elevation of serum VEGF has been observed in patients with different types of cancer [50]. The rationale for the increased serum VEGF in cancer patients can be explained by an enhanced thrombin activation of platelets resulting in the release of the IP growth factors. The diagnostic relevance of platelet VEGF has been explained in the detection of various cancers, sometimes even better than the classical tumor markers. Serum VEGF, which reflects the total IP concentration, was found to be more useful test than tissue VEGF for the prognosis of HCC [51].

Diagnostic and prognostic role of VEGF in HCC has been extensively explored. Higher serum VEGF per platelet count was observed in patients with HCC [49]. Serum VEGF correlated with HCC progression and also predicted treatment outcome [49, 52]. Results obtained from the preparation of isolated platelet extracts (IP VEGF) also corresponded to the conclusions drawn from the serum VEGF [48]. Overall, an increase in serum VEGF is a poor prognostic indicator in HCC patients undergoing therapies like radiofrequency ablation (RFA), TACE, and hepatic resection [53, 54]. One study that was carried out in western population came up with a contradictory result showing no predictive significance of serum VEGF in the adverse outcome of HCC in patients undergoing liver transplantation [55].

Serum VEGF, derived from platelets, is also considered a potential biomarker of sorafenib efficacy in HCC not amenable to locoregional therapy, including surgery, RFA, or TACE. Sorafenib is a small tyrosine kinase inhibitor that targets VEGF receptor (VEGFR), PDGF receptor-*β*, and Raf kinase. A meta-analysis pooled from 9 different studies concluded that high VEGF concentrations after sorafenib administration would suggest poor survival and poor clinical efficacy [56]. Strikingly, another study observed an association of lower serum VEGF concentration with a higher disease control rate but not improved PFS and OS after sorafenib treatment [57]. Since VEGFR-2 is a key target of sorafenib treatment, evaluation of platelet VEGF requires more precise and careful consideration as a predictive tool in patients undergoing sorafenib treatment.

## 8. Implicating the Profibrotic Platelet-Derived Growth Factor and Transforming Growth Factor

Evidence from rodents has been translated in human studies substantiating the profibrotic effect of transforming growth factor beta-1 (TGF-*β*1) and platelets derived growth factor-BB (PDGF-BB). Serum concentration of PDGF-BB was able to reflect degree of liver damage and degree of liver fibrosis in patients with chronic hepatitis B (CHB) [58]; PDGF level was positively correlated with the degree of liver damage. Another diametrically opposite in-human evidence came up with a significant decrease in serum PDGF-BB with increased fibrosis proposing the depleted serum PDGF-BB as a biomarker for the assessment of fibrosis stage in patients with CHB [59]. The latter finding explains the prohealing action of PDGF-BB in liver pathophysiology. No documented evidence on the diagnostic role of PDGF-BB in HCC was found; however, higher concentrations of PDGF-BB in the serum of patients with HCC under sorafenib treatment tended to extend survival benefits [60].

Megakaryocytes are the major sites of production and storage of TGF-*β*1 [61]; TGF-*β*1 is incorporated into the alpha granules of the platelets. Some functions of TGF- *β*1 in the liver include stimulation of extracellular matrix and production of fibrous tissue. TGF-*β*1 concentration in serum reflects the total platelet TGF-*β*1 [62]. Contrary to its stimulatory effects on other cells, TGF-*β*1 exerts a negative effect on hepatocyte proliferation via induction of apoptotic mechanisms [63]. An elevated concentration of serum TGF-*β*1 is documented in patients with CHC that is presumed to have some roles in the pathogenesis and chronicity of these diseases [63]. In a nested case-controlled study, Watanabe et al. reported a strong association between a low serum TGF-*β*1 concentration and a higher risk of incidence HCC [64]. Their result obtained from a large population indicates the predictive potential of serum TGF-*β*1 in identifying high-risk patients who are likely to develop HCC, especially among HCV-positive patients. Serum TGF-*β*1 was not only elevated in patients with HCC but also correlated with the tumor progression [65]. Intriguingly, serum TGF-*β*1 concentrations improved the detection of AFP-negative HCC in high-risk patients [66]. In another study, the biological function of TGF-*β*1 was evaluated by analyzing downstream molecules involved in the progression of HCC in a large cohort of HCC patients in different disease stages; the study concluded that, rather than indicating advanced HCC, TGF-*β*1 corresponds to intrinsic biological properties of the tumor, affecting progression and the clinical outcome of the disease [67]. Serum TGF-*β*1 concentration has also been implicated to predict outcome in HCC patients treated with sorafenib; high pretreatment serum TGF-*β*1 levels were linked with poor PFS and OS. There are still ample possibilities for investigating and validating the diagnostic or prognostic implications of these profibrotic growth factors in HCC.

## 9. Epidermal Growth Factor and Fibroblast Growth Factor

The megakaryocytes synthesized epidermal growth factor (EGF) is largely present in the alpha granules of human platelets [68]. EGF is directly associated with the inflammatory microenvironment and regulation of HCC proliferation and migration and enhances the metastatic potential of cancer cells. Serum level of EGF was found elevated in HCC patients, and it was useful in identifying HCC from CHC patients [69]. Serum EGF also indicated HCC progression, and an elevated serum EGF concentration was a predictor of poor OS [70]. Tanabe et al. investigated EGF in the serum of G/G and A/A genotype (polymorphism in the EGF gene) in patients with cirrhosis; the G/G genotype group, with a higher risk of HCC development, showed significantly elevated serum EGF concentration. The study interpreted an association between EGF gene polymorphism and HCC progression in liver cirrhosis via modulation of EGF levels [71].

Fibroblast growth factors (FGF), multifunctional proteins, possess angiogenic property stronger than VEGF or PDGF [72]. Stromal cells produced FGF is largely reserved in the granules of platelet [73]. Some evidence has highlighted the pivotal role of FGF in HCC occurrence and progression [74]. The serum level of FGF-2 was significantly elevated in patients with liver cirrhosis, CH, and HCC compared with those of healthy controls [75]. A study conducted to determine the activities of several serum growth factors in living donor liver transplantation observed a marked increase in serum FGF-2 concentrations, with a high statistical power, in patients with HCC recurrence [76]. Chen et al. simultaneously evaluated 39 serum cytokines in 2 cohorts of HCC patients after radical resection; along with 5 other cytokines, serum FGF-2 better predicted the DFS [77].

## 10. Conclusions and Future Directions

**Figure 1:**
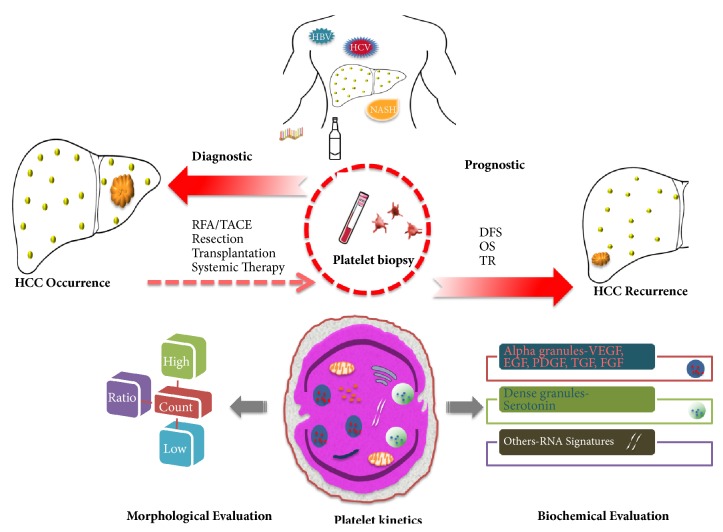
**Schematic representation of platelet-based diagnostic and prognostic model in HCC.** Platelet may serve as a liquid biopsy in patients with underlying liver disease to detect or predict HCC occurrence. Posttreatment platelets, after locoregional therapy, resection, transplant, or systemic therapy, may also enable monitoring the prognosis including DFS, OS, and TR. The morphological abnormalities of platelets including thrombocytosis, thrombocytopenia, or platelet ratio with lymphocyte/neutrophil have been used to evaluate the diagnosis or prognosis of HCC. Platelet-derived growth factors (VEGF, EGF, PDGF, FGF, and serotonin) are differentially expressed and correlated with diagnosis or prognosis of HCC. Other emerging platelet-based tools like RNA signatures are yet to be tested in patients with HCC. (HBV, hepatitis B virus; HCV, hepatitis C virus; NASH, nonalcoholic steatohepatitis; HCC, hepatocellular carcinoma; DFS, disease-free survival; TACE, transarterial chemoembolization; RFA, radiofrequency ablation; OS, overall survival; TR, treatment response; VEGF, vascular endothelial growth factor; EGF, epidermal growth factor; PDGF, platelet-derived growth factor; FGF, fibroblast growth factor).

Possibilities are increasingly apparent for building platelet-based diagnostic or prognostic system in HCC. With all the above-mentioned platelet-based information, the multifaceted platelets may serve as a minimally invasive form of liquid biopsy enabling a valid test for detecting and monitoring the prognosis of HCC (Figure 1).

Differential expression of platelet alpha or dense granule proteins, such as IP serotonin and IP VEGF, in HCC patients has influenced the horizon of platelet-based diagnostics. Concurrently, divergent results on the prognostic potential of platelet growth factors in HCC may relate to multiple factors. As discussed in the manuscript, the platelet growth factors were typically assayed in serum. Serum analysis may not include entire analytes found in the granules of platelets; some IP growth factors are presumed to be released into the serum during agonist (thrombin or ADP) stimulation during serum clot formation, but a substantial amount still remains associated with the platelets and is likely lost with the hematocrit [78, 79]. Thus, isolation of platelet fraction and analyzing its content remain crucial to specifically assess the concentration of IP growth factors and avoid the complication of misinterpretation when using the serum samples. Furthermore, preparation of pure platelet fraction and analysis of IP proteins are a highly sensitive procedure leading to frequent artifacts. Starlinger et al. have discussed and demonstrated how variants of blood processing affect the clinical monitoring of platelet-stored angiogenic growth factors [80]. Consequently, optimization of platelet fraction remains the foundation for accuracy in the analyses of IP growth factors.

The paradoxical platelet kinetics has made it more complex to mechanistically comprehend its property as a diagnostic marker in HCC. Platelet biomarkers were not assessed at a uniform time point in the aforementioned studies. Platelets are believed to affect the liver pathophysiology in disease-specific, stage-specific, and site-specific manner [9]; we previously reported how IP VEGF-A reflects tumor character at one stage and liver regeneration at the other [48]. Although pretreatment platelets are palatable for diagnostic or prognostic system, the platelets from posttreatment period warrant a high caution; for example, the platelet fraction from the immediate postresection patient may be influenced by inflammatory environment or active liver regeneration [44]. The time point must be taken into account when using platelet as a diagnostic or prognostic tool in HCC, and this also explains the contradictory results observed in different studies with the identical biomarkers.

The introduction of tumor-educated platelets (TEP) in platelet-based cancer diagnostic is another intriguing progress. Based on platelet RNA analyses, Best et al. distinguished cancer patients from healthy subjects with greater than 95% accuracy [81]. They have demonstrated a novel possibility of using TEP RNA as a noninvasive, blood-based liquid biopsy, a marker that allowed efficient, rapid, and comprehensive molecular characterization of tumors. In their previous study, with the aid of particle-swam optimization algorithms and RNA-seq of TEP, they were able to generate the diagnostic TEP that enabled identifying patients with nonsmall-cell lung cancer from individuals without cancer, including those having inflammatory conditions [82]. This exhilarating early discovery of diagnostic TEP needs to be followed by studies of larger cohorts of individuals with specific tumor types, able to recapitulate the results. Platelet RNA signatures have emerged with the possibility of potential revolution in cancer diagnostics [83, 84]. Several platelet-based candidate entities are trying to establish themselves in the diagnostic and prognostic of HCC; the implementation of TEP in HCC could be highly instrumental in interrogating its reliability in the realm of cancer diagnostic.

Kanikarla-Marie et al. describe “circulome” as an entity that integrates all the circulating factors in the bloodstream [85]. Besides secreting proteins and other components into it, platelets also take up molecules from the circulome. Platelets, platelet exosomes, and tumor cell-induced platelet activation participate in critical events of tumorigenesis. The phenotypic changes of platelets are influenced by the interaction with tumor phenotype. A better understanding of the phenotypic alterations of platelets could provide highly valuable information in cancer diagnostics.

Unlike several other malignant diseases, diagnosis of HCC does not necessarily require a tissue biopsy or histological verification. Platelet can offer itself as a liquid biopsy with a potential revolution in HCC diagnostics and prognostics. Substantial progress has been made in HCC biomarkers discovery, but only a modest number of markers have shown their relevance in clinical settings. Moreover, the existing classical biomarkers are less promising in predicting prognosis and HCC recurrence. Platelets patrolling the injured liver, interacting with hepatic sinusoidal endothelium, and influencing effector cell recruitment and activation no more remain the mystery [9]. We are witnessing several potential avenues of platelet-based cancer diagnostic; however, the complex and paradoxical properties of platelets outlined above necessitate building a more solid foundation before platelet takes the lead in diagnostic and prognostic evaluation of HCC.
